# Time-Controlled Adaptive Ventilation Does Not Induce Hemodynamic Impairment in a Swine ARDS Model

**DOI:** 10.3389/fmed.2022.883950

**Published:** 2022-05-17

**Authors:** Mickael Lescroart, Benjamin Pequignot, Laurent Bitker, Héloïse Pina, N'Guyen Tran, Jean-Louis Hébert, Jean-Christophe Richard, Bruno Lévy, Matthieu Koszutski

**Affiliations:** ^1^CHRU Nancy, Service de Médecine Intensive et Réanimation, Hôpital Brabois, Vandœuvre-lès-Nancy, France; ^2^INSERM U 1116, Groupe Choc, Équipe 2, Faculté de Médecine, Vandœuvre-lès-Nancy, France; ^3^Université de Lorraine, Faculté de Médecine, Nancy, France; ^4^Service de Médecine Intensive - Réanimation, Hôpital de la Croix-Rousse, Hospices Civils de Lyon, Lyon, France; ^5^Université de Lyon, Université Claude Bernard Lyon 1, Lyon, France; ^6^CHRU de Nancy, Département D'Anatomie Pathologique, Laboratoires de Biologie Médicale et de Biopathologie, Hôpital Brabois, Vandœuvre-lès-Nancy, France; ^7^Ecole de Chirurgie, Faculté de Médecine, Université de Lorraine, Nancy, France; ^8^Université Paris XI, Institut de Cardiologie, Groupe Hospitalier Pitié-Salpêtrière, Paris, France

**Keywords:** mechanical ventilation, ARDS, TCAV, APRV, hemodynamic, heart-lung interactions

## Abstract

**Background:**

The current standard of care during severe acute respiratory distress syndrome (ARDS) is based on low tidal volume (VT) ventilation, at 6 mL/kg of predicted body weight. The time-controlled adaptive ventilation (TCAV) is an alternative strategy, based on specific settings of the airway pressure release ventilation (APRV) mode. Briefly, TCAV reduces lung injury, including: (1) an improvement in alveolar recruitment and homogeneity; (2) reduction in alveolar and alveolar duct micro-strain and stress-risers. TCAV can result in higher intra-thoracic pressures and thus impair hemodynamics resulting from heart-lung interactions. The objective of our study was to compare hemodynamics between TCAV and conventional protective ventilation in a porcine ARDS model.

**Methods:**

In 10 pigs (63–73 kg), lung injury was induced by repeated bronchial saline lavages followed by 2 h of injurious ventilation. The animals were then randomized into two groups: (1) Conventional protective ventilation with a VT of 6 mL/kg and PEEP adjusted to a plateau pressure set between 28 and 30 cmH_2_O; (2) TCAV group with P-high set between 27 and 29 cmH_2_O, P-low at 0 cmH_2_O, T-low adjusted to terminate at 75% of the expiratory flow peak, and T-high at 3–4 s, with I:E > 6:1.

**Results:**

Both lung elastance and PaO_2_:FiO_2_ were consistent with severe ARDS after 2 h of injurious mechanical ventilation. There was no significant difference in systemic arterial blood pressure, pulmonary blood pressure or cardiac output between Conventional protective ventilation and TCAV. Levels of total PEEP were significantly higher in the TCAV group (*p* < 0.05). Driving pressure and lung elastance were significantly lower in the TCAV group (*p* < 0.05).

**Conclusion:**

No hemodynamic adverse events were observed in the TCAV group compared as to the standard protective ventilation group in this swine ARDS model, and TCAV appeared to be beneficial to the respiratory system.

## Introduction

Acute Respiratory Distress Syndrome (ARDS) is a life-threatening condition due to a lung injury that can result from numerous causes (e.g., infectious, toxic, or inflammatory). Its mortality raises up to 50% in the most severe cases (1).

ARDS treatment is based on protective mechanical ventilation, prone positioning, neuromuscular blockade or VV-ECMO (2). The current standard of care is based on the limitation of ventilator-induced lung injury (VILI) by reducing the insufflated tidal volume (V_T_) to 6 mL/kg of predicted body weight (PBW) and by maintaining driving pressure (ΔP) below 15 cmH_2_O (3, 4). As positive end expiratory pressure (PEEP) can provide both lung recruitment and overdistension, it can lead to an increase in pulmonary blood pressure (PBP) (5, 6). An alternative strategy is the time-controlled adaptive ventilation (TCAV), a specific combination of settings applied to set the airway pressure release ventilation (APRV) mode. Initially reported by Habashi et al., TCAV reduces lung injury in both experimental and clinical studies (7–9). TCAV is based on delivering a continuous inspiratory positive airway pressure (CPAP) phase (P_high_), followed by a brief expiratory release phase (T_low_) (10).

A significant concern is the hemodynamic effect of an increase in intrathoracic pressure leading to a decrease in cardiac output (6, 11). Our hypothesis is that TCAV, that results in higher intra-thoracic pressures due to the prolonged inspiratory phase, can lead to harmful heart-lung interactions. The main objective of our study was to compare hemodynamics during the first hour of TCAV or conventional protective ventilation in a porcine ARDS model.

## Methods

The present study was conducted in accordance with the ARRIVE consensus guideline for reporting animal experimental studies (12).

### Ethics

All experiments were reviewed and approved by the Nancy University Ethics Committee for Animal Experimentation (APAFIS Number 2020082407561244). The procedure for the care and sacrifice of the study animals was in accordance with the European Community Standards on the Care and Use of Laboratory Animals.

### Animal Preparation

Animals were fasted overnight with free access to water. All the pigs were of male sex with a median weight of 67 kilograms. Intramuscular premedication was performed with ketamine (1.5 mg/kg, Warner Lambert, Nordic, AB Solna, Sweden) before transportation to the experiment facility. Sedation was deepened with propofol (2.5 mg/kg, B. Braun, Melsungen, Germany) *via* an ear vein cannula. After being placed in a supine position, animals were intubated with a 7.5-mm internal diameter endotracheal tube (ETT). Anesthesia was maintained with a continuous infusion of midazolam 5 mg/h and sufentanyl 20 μg/h. Depth of anesthesia was assessed regularly by checking on movements and hemodynamic response to a painful stimulus. Muscle paralysis was then maintained with a continuous infusion of cisatracurium (0.5 mg/kg/h) (GlaxoSmithKline, Marly-le-Roi, France) throughout the experiment. Pigs were connected to the ventilator (Dräger Evita Infinity V500, Lübeck, Germany), with the baseline settings adjusted to the following levels: V_T_,7 mL/kg; respiratory rate (RR), 22 breaths/min; PEEP, 5 cmH_2_O; fraction of inspired oxygen (FiO_2_),100%. Automatic tube compensation (ATC) was adjusted to 100%. The ventilator settings were then adjusted to pH > 7.35 and PaCO_2_ between 40 and 45 mmHg.

### Hemodynamic Monitoring

Measurements were performed at the following successive periods: after intubation and catheters placement at basal state (T_B_), after ARDS induction with saline lavages and injurious mechanical ventilation (T_0_), and at 15 min (T_15_) and 60 min (T_60_) following randomization to either conventional protective ventilation or TCAV (Supplementary Figure 1). A pulmonary artery catheter (Swan-Ganz, Edwards Lifesciences, Irvine, USA) was inserted *via* the left internal jugular vein for measuring PBP, pulmonary artery wedge pressure (PAWP), right atrial pressure (RAP) and mixed venous oxygen saturation (SVO_2_). The pressure transducer was positioned at the level of the right atrium. A conductance catheter (Transonic Systems Inc., Ithaca, USA) was inserted into the left ventricle *via* the left carotid artery for simultaneous registration of both instantaneous high-fidelity left ventricular pressure (PLV) and instantaneous left ventricular volume. Central aortic pressure (ABP) was assessed by a high-fidelity pressure catheter (HIFI) (Transonic Systems Inc., Ithaca, USA) percutaneously inserted *via* the femoral artery into the descending thoracic aorta. The catheters were inserted under fluoroscopy. The right carotid artery was dissected, and a Transit Time Flow probe (Transonic Systems Inc., Ithaca, USA) was secured around it. Data were computed using a designated analysis program (IOX 2.4.2.6^®^, EMKA Technologies, France). The signals were recorded continuously at a sampling rate of 2,000 Hz. A period of 2 h was required for the calibration and the correct positioning of the probes, assessed by fluoroscopy and chest X Ray. The core body temperature was measured *via* a rectal probe and maintained between 37 and 38° by a warming blanket system.

### Respiratory Monitoring

Airway pressure (P_aw_) was continuously registered by a probe set on the ventilator Y-piece. The esophageal pressure (P_es_) was assessed by an esophageal balloon (BA-A-008 probe, MBMed, Argentina) positioned with fluoroscopy and inflated up to 4 mL. The correct positioning of the devices was checked by using the Baydur manoeuver (13). Transpulmonary pressure (P_L)_ was calculated in absolute value, as follows: P_L_ = P_aw_ – P_es_. ΔP_L_ is defined as the difference between P_Lend−insp_ and P_Lend−exp_. The absolute value of P_L_ reflects the local pressure in the dependent lung regions, adjacent to the esophageal balloon, independently of the mediastinal structures (14). Elastance of the respiratory system (El_RS_) was assessed by: El_RS_ = ΔP_aw_/V_T_. The elastance ratio (ER) was calculated as follows: ER = El_L_/El_RS_, i.e., the lung elastance (El_L_) to total respiratory system elastance ratio (15). Inspiratory transpulmonary pressure based on elastance ratio (P_L_Er) reflects the local pressure in the non-dependent lung regions (16). It was calculated as follows: P_L_Er = P_aw_ x ER. End inspiratory and end expiratory P_L_ were measured after a 5-s airway occlusion of the ventilator circuitry. Data were computed using a designated analysis program with sampling rate of 2,000 Hz (IOX 2.4.2.6^®^, EMKA Technologies, France). In TCAV, total PEEP was measured during a 5-s occlusion period at the end of expiration.

End-tidal carbon dioxide (EtCO_2_) was monitored for assessing the PaCO_2_-EtCO_2_ gradient and estimate the physiologic dead space as described by Enghoff's modification of the Bohr equation: $\frac{VD}{VT}$ = $\frac{PaCO2\;-\;EtCO2\;}{PaCO2}$ where VD is the dead space volume (mL), V_T_ is tidal volume (mL), EtCO_2_ is the end tidal expiratory CO_2_ (mmHg), and PaCO_2_ (mmHg) is the systemic arterial CO_2_ pressure (17).

### Electrical Impedance Tomography

An electrical impedance tomography (EIT) electrode belt, which carries 16 electrodes with an inter-electrode distance of 40 mm, was placed around the thorax in the fifth intercostal space, and one reference electrode was placed on the animal's abdomen (PulmoVista 500, Dräger Medical, Lübeck, Germany). The measures of EIT were averaged over five respiratory cycles and the images were divided into four regions of interest (ROI): ROI 1 being the most ventral, to ROI 4, being the most dorsal. Results are expressed as the percentage of total tidal volume ventilation in the four ROIs (18, 19). The regional compliance was calculated in the four ROIs as follows: $RC_{ROI}=\frac{VT\;x\;ROI\;}{\Delta Paw}$ expressed in mL/cmH_2_O.

### ARDS Induction

Induction of a double hit lung injury was performed by 4 repeated lung lavages for a total of 30 mL/kg warm 0.9% saline solution intratracheally at 38.5°C. The lung was filled up to the endotracheal tube and fluid was drawn from the airways after 2 min *via* a tracheal aspiration. During the bronchoalveolar lavage, all the animals developed a profound desaturation with SpO_2_ < 60% without any bradycardia or life-threatening hemodynamic alteration. This was followed by 2 h of injurious ventilation with PEEP 0 cmH_2_O and inspiratory pressure of 40 cmH_2_O, RR 10/min, inspiratory to expiratory time ratio (I:E) of 1:1 (20). The FiO_2_ was set at 1.0, providing an additional mechanism of lung injury (21). Of note, mechanical power of mechanical ventilation transferred to the respiratory system was estimated at 41 J/min, by applying the equation proposed by Louis et al. (22). The animals received a continuous intravenous infusion of normal saline at 10 mL/Kg/h during lung injury induction, and 2 mL/Kg/h during the study period.

### Interventions and Study Groups

After the induction of ARDS, animals were randomly allocated to one of the following two groups:

- *Conventional protective group (n* = *5):* with V_T_ 6 mL/kg, PEEP adjusted to reach a plateau pressure of 28 to 30 cmH_2_O, RR 25 bpm, I:E 1:2.
- *TCAV group (n* = *5):* P_high_ set between 27 and 29 cmH_2_O, P_low_ at 0 cmH_2_O, T_low_ set to terminate at 75% of the expiratory flow peak, T_high_ at 3–4 s, and I:E > 6:1.

## Statistical Analyses

Given the small sample size, all results are expressed as median and interquartile range (IQR). Baseline and T_0_ measurements were compared by using the non-parametric Friedman test for analysis of variance by ranks. Respiratory and hemodynamics values between the two groups at T_0_, T_15_, and T_60_ were compared by using mixed effects regression models for evaluating the association of variables of interest (fixed effects) with the dependent variable, using the animal number as random effect to account for the repetition of regional measurements in each animal, and the lung level as a random slope. Multicollinearity and interactions were systematically evaluated in multivariate models; in the case of a significant interaction, a *post-hoc* analysis using pairwise comparison adjusted for the repetition of statistical tests was performed using the Tukey method. In the case of *post-hoc* multiple comparisons to a single reference level, we used the Dunnett adjustment method. All statistical analyses were with a significance level of 0.05 and performed using R version 4.0.1 for MacOS^®^ (https://www.r-project.org/, accessed March 2020).

## Results

### Effect of Experimental ARDS on Respiratory Mechanics and Hemodynamics

Thirteen pigs were involved in the experiment. Ten pigs were included into the final analyses. Two pigs developed an early hemorrhagic shock, and one pig developed a refractory ventricular fibrillation at the time of the left ventricular catheter insertion before randomization.

Respiratory and hemodynamic parameters at baseline and after ARDS induction are summarized in Supplementary Table 1. At T_0_ (after ARDS induction) both El_L_ [32 cmH_2_O/L (29–33)] and PaO_2_/FiO_2_ ratio [99 (88–115)] were consistent with a severe ARDS.

### Effect of Ventilation Strategies on Hemodynamics

All the results related to hemodynamics are presented in Table 1 and Supplementary Table 2. There were no significant between group differences at T_0_ for the main hemodynamic parameters: heart rate (HR), cardiac output (CO), ABP, PBP, RAP, and pulmonary vascular resistance (PVR). The only significant difference was observed for the left ventricle (LV) isovolumic relaxation time constant (Tau) and LV maximal rate of pressure rise (LV + dP/dtmax) values, which reached higher levels in the conventional protective ventilation population at T_0_ but also at T_15_ and T_60_ (*p* < 0.05, no interaction was detected in multivariate analysis). There was no between-group difference at T_60_ for HR, CO, ABP, PBP, RAP and PVR. There was no between group difference in lactate values at T_60_ between the TCAV group [1.1 mmol/L (1.0–2.1)] vs. 1.5 (1.5–1.7) in the conventional protective group (*p* = 0.06).

**Table 1:** Hemodynamic characteristics.

	**TCAV (*n* = 5)**	**Conventional protective ventilation (*n* = 5)**	**Effect of group**	**Effect of time**	**Group × time**
**Heart rate (bpm)**			*p* = 0.3	*p* = 0.2	*p* = 0.4
T0	122 (121 to 134)	136 (135 to 137)			
T15	135 (134 to 136)	132 (129 to 141)			
T60	133 (130 to 139)	135 (134 to 138)			
**Mean aortic blood pressure (mmHg)**			*p* = 0.9	*p* = 0.7	*p* = 0.7
T0	104 (92 to 104)	85 (83 to 102)			
T15	100 (95 to 105)	90 (85 to 110)			
T60	104 (90 to 109)	90 (76 to 95)			
**Mean pulmonary blood pressure (mmHg)**			*p* = 0.4	*p* = 0.3	*p* = 0.3
T0	39 (35 to 40)	40 (34 to 42)			
T15	38 (33 to 40)	32 (27 to 42)			
T60	37 (36 to 38)	40 (30 to 46)			
**Pulmonary vascular resistance (U Woods)**			*p* = 0.3	*p* = 0.1	*p* = 0.3
T0	2.6 (2.3 to 3.5)	2.8 (2.4 to 3.0)			
T15	2.7 (2.8 to 3.1)	2.5 (2.3 to 3.2)			
T60	3.1 (3.0 to 3.5)	2.6 (2.2 to 3.8)			
**Right atrial pressure (mmHg)**			*p* = 0.4	*p* = 0.7	*p* = 0.3
T0	9 (9 to 10)	10 (9 to 11)			
T15	11(10 to 11)	11 (10 to 13)			
T60	9 (9 to 10)	9 (8 to 11)			
**PAWP (mmHg)**			*p* = 0.3	*p* = 0.1	*p* = 0.08
T0	13 (13 to 14)	14 (13 to 17)			
T15	14 (13 to 16)	13 (12 to 14)			
T60	12 (10 to 14)	11 (10 to 12)			
**Cardiac output (L.min** ^**−1**^ **)**			*p* = 0.3	*p* = 0.1	*p* = 0.06
T0	8.7 (6.8 to 9.9)	6.5 (6.0 to 9.3)			
T15	8.1 (8.0 to 9.7)	8.7 (8.0 to 9.7)			
T60	7.6 (5.5 to 8.6)	7.6 (6.9 to 11.5)			
**LV Tau 1/e (ms)**			*p* < 0.05	*p* = 0.1	*p* = 0.3
T0	20.6 (18.0 to 22.0)	13.5 (10.1 to 15.8)			
T15	16.0 (15.8 to 21.3)	13.6 (9.7 to 15.4)			
T60	20.1(16.0 to 20.9)	15.6 (14.1 to 17.9)			
**LV –dP/dtmax (mmHg.s-1)**			*p* = 0.3	*p* = 0.5	*p* = 0.8
T0	−1,719 (−2,397 to −1,545)	−2,987 (−3,000 to −1,984)			
T15	−1,972 (−2,060 to −1,785)	−2,100 (−2,527 to −1,115)			
T60	−2,048 (−2,150 to −1,695)	−2,489 (−2,878 to −1,855)			
**LV +dP/dtmax (mmHg.s** ^**−1**^ **)**			*p* < 0.05	*p* = 0.4	*p* = 0.3
T0	1,738 (1,661 to 4,772)	3,969 (3,460 to 4,179)			
T15	1,609 (1,494 to 4,737)	3,746 (1,848 to 6,044)			
T60	1,604 (1,483 to 5,038)	4,404 (4,334 to 6,816)			
**LV +/–dP ratio**			*p* = 0.4	*p* = 0.1	*p* = 0.2
T0	1.32 (0.65 to 2.74)	1.91 (1.35 to 2.08)			
T15	1.19 (0.75 to 2.65)	2.79 (2.39 to 2.80)			
T60	2.13 (0.87 to 2.45)	2.65 (1.32 to 3.26)			
**Total fluid loading (mL)**			*p* = 0.3	*p* = 0.1	*p* = 0.3
T0	1,675 (1,650 to 1,825)	1,660 (1,570 to 1,830)			
T15	1,710 (1,680 to 1,860)	1,700 (1,610 to 1,860)			
T60	1,820 (1,780 to 1,970)	1,810 (1,780 to 1,960)			

*The analysis used all data collected in both groups at the 3 study time points, using a mixed effects linear regression with study group and study time point as independent variables, and animal identification number as the random effect. Interaction of time with study group was systematically checked for. If no interaction was identified, the p-value of the effect of Group and Time are given, respectively. In case of a significant interaction, a pairwise post-hoc multiple comparison was performed to compare groups at each time points on the one side, and compare T15 and T30 to T0 in each group, on the other. TCAV, Time controlled adaptative ventilation; PAWP, Pulmonary artery wedge pressure; LV +dP/dtmax and LV -dP/dtmax, minimum and maximum rate of pressure change in the left ventricle; LV dP ratio, represent catecholaminergic impregnation and was calculated as the ratio of -dP/dtmax and +dP/dtmax; LV Tau, Isovolumic relaxation constant. T0: After ARDS induction; T15: 15 min after start of study; T60: 60 min after start of study; Data are presented as median (25th−75th percentile)*.

**Figure 1:**
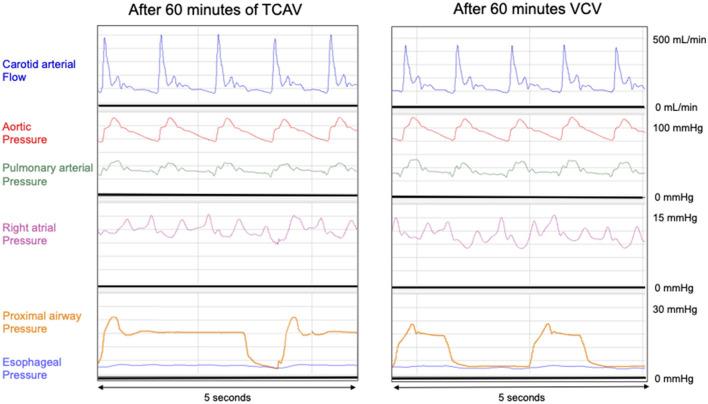
Polygraphic recordings between two groups at T_60_ of the main hemodynamic and respiratory outcomes. VCV: Conventional protective group with VT 6 ml.kg^−1^, PEEP 10 cmH_2_O, RR 25 bpm, I:E 1:2. TCAV: Phigh 27 cmH_2_O, Plow at 0 cmH_2_O, Tlow 0.4 s, Thigh 4.

Polygraphic recordings between two groups at T_60_ of the main hemodynamic and respiratory outcomes are presented in Figure 1.

### Effects of the Ventilation Strategies on Respiratory Mechanics

All the results related to respiratory parameters are presented in Table 2 and Supplementary Table 3. There were no significant differences in the respiratory parameters between the TCAV and conventional protective groups at T_0_ except for pH (*p* < 0.05).

**Table 2:** Respiratory characteristics.

	**TCAV (*n* = 5)**	**Conventional protective ventilation** **(*n* = 5)**	**Effect of group**	**Effect of time**	**Group × time**
**VT (mL/kg)**			-	-	*p* < 0.05
T0	5.9 (5.5–6.0)	5.9 (5.9–6.0)			
T15	6.9 (6.2–7.2)	5.9 (5.7–6.1)			
T60	7.4 (6.4–7.8)^a^	6.1 (5.8–6.2)^b^^c^			
**RR (.min** ^**−1**^ **)**			-	-	
T0	24 (23–26)	25 (24–26)			*p* < 0.05
T15	20 (18–20)^a^	27 (26–28)^b^^c^			
T60	20 (18–20)^a^	27 (26–28)^b^^c^			
**PEEPt (cmH**_**2**_ **O)**			-	-	*p* < 0.05
T0	5 (5–6)	5 (5–6)			
T15	11 (10–13)^a^	11 (11–11) ^b^			
T60	14 (14–15)^a^	11 (11–11)^b^^c^			
**ΔP**_**AW**_ **(cmH**_**2**_**O)**			-	-	*p* < 0.05
T0	19 (18–21)	20 (19–21)			
T15	14 (13–15)^a^	18 (18–19)^b^^c^			
T60	13 (11–14)^a^	18 (18–19)^b^^c^			
**ΔP**_**L**_ **(cmH**_**2**_**O)**			-	-	*p* < 0.05
T0	15 (14–16)	17 (15–18)			
T15	11 (8–11)^a^	16 (15–19)^c^			
T60	10 (7–11)^a^	15 (12–16)^c^			
**El**_**L**_ **(cmH**_**2**_**O.L**^**−1**^**)**			-	-	*p* < 0.05
T0	41 (40–41)	40 (37–41)			
T15	25 (19–26)^a^	42 (41–45)^c^			
T60	22 (15–23)^a^	40 (39–42)^c^			
**PaCO**_**2**_ **(mmHg)**			*p* = 0.3	*p* = 0.5	*p* = 0.4
T0	43 (35–44)	48 (44–49)			
T15	44 (38–45)	43 (41–49)			
T60	39 (37–45)	46 (38–54)			
**PaO**_**2**_**/FIO**_**2**_ **(mmHg)**			*p* = 0.5	*p* < 0.05	*p* = 0.3
T0	88 (44–99)	100 (98–115)			
T15	140 (95–200)	101 (80–117)			
T60	135 (100–219)	117 (75–180)		^#^	

*The analysis used all data collected in both groups at the 3 study time points, using a mixed effects linear regression with study group and study time point as independent variables, and animal identification number as the random effect. Interaction of time with study group was systematically checked for. If no interaction was identified, the p-value of the effect of Group and Time are given, respectively. In case of a significant interaction, a pairwise post-hoc multiple comparison was performed to compare groups at each time points on the one side, and compare T15 and T30 to T0 in each group, on the other*.

# *p < 0.05 compared to T0 at the time point (no interaction with study group)*.

a *p < 0.05 compared to T0 in the TCAV group in multiple comparison*.

b *p < 0.05 compared to T0 in the conventional protective ventilation group in multiple comparison*.

c *p < 0.05 compared to the TCAV group at this time point in multiple comparison*.

*TCAV, Time controlled adaptative ventilation; VT, Tidal volume; RR, Respiratory rate; PEEPtot, Positive End Expiratory Pressure total; ΔP_AW_, driving pressure, difference in airway pressure at end-inspiration (plateau pressure) and end-expiration (total PEEP); ΔP_L_, difference in transpulmonary inspiratory pressure at end-inspiration and end-expiration; El_L_, lung elastance; T0, After ARDS induction; T15, 15 min after start of study; T60, 60 min after start of study; Data are presented as median (25th−75th percentile)*.

Respiratory rate was significantly lower at T_60_ in the TCAV group compared to the conventional protective group (*p* < 0.05). Levels of total PEEP were significantly higher in the TCAV group at T_60_ (*p* < 0.05). Mean airway pressure was significantly higher in the TCAV group at T_15_ and T_60_ (*p* < 0.05). The ΔP_aw_ was significantly lower in the TCAV group at T_15_ and T_60_ (*p* < 0.05). V_T_ in the TCAV group significantly differed from conventional protective group at T_60_: 7.4 mL/kg (6.4–7.8) in the TCAV group vs. 6.1 mL/kg (5.8–6.2) in the conventional protective group (*p* < 0.05). Elastance of the lung at T_15_ and T_60_ was significantly lower in the TCAV group (*p* < 0.05). PaO_2_/FiO_2_ increased in both groups at T_60_ without significant differences between the two groups. During the study period PaCO_2_ did not differ significantly. The regional compliance in the mid-ventral and mid-dorsal regions (RC_ROI_ 2 and RC_ROI_ 3) was significantly higher at T_60_ in the TCAV group (*p* < 0.05) (Supplementary Table 4).

### Fluid Loading and Vasopressors

The total fluid loading was of 1,675 mL (1,650–1,825) in the TCAV group and of 1,660 ml (1,570–1,830) in the VCV group (*p* = 0.3) and no norepinephrine was infused during the study period (Table 2).

## Discussion

The main result of the present study is that TCAV did not significantly impact hemodynamics, despite the increase in intrathoracic pressures. Additionally, TCAV improved the lung elastance after only 1 h of ventilation.

### ARDS Model

Saline lavages followed by 2 h of injurious mechanical ventilation is a well-established model for inducing ARDS. It provides a highly reproducible and significant homogenous alteration of the PaO_2_/FiO_2_, El_L_, and the dead space volume. ER was 0.8 after ARDS induction, indicating specific lung involvement for El_RS_ alteration without chest wall participation (16). This method provided a triple-hit lung injury: saline lavages leads to surfactant depletion, 100% oxygen delivery can lead to denitrogenation and injurious ventilation provides both barotrauma and volotrauma (23).

### Hemodynamic Assessment of TCAV

In our work, TCAV was not associated with a hemodynamic impairment compared to standard ventilation. Regarding the right ventricular function, there were no elements suggestive of right ventricle failure, as right atrial pressure values remained low in both groups and the cardiac output was stable during the study period. Even if higher intrathoracic pressures can impair hemodynamics, changes in lung physiology can have beneficial consequences on the right ventricle and thus on hemodynamics. As pulmonary vascular resistance relates to lung volume, higher intrathoracic pressures could be in fact associated with an increase in FRC and thus a reduction in PVR (24). Sharpey-Shafer et al. reported in 1965 that a “square wave” response of the arterial pressure to the Valsalva maneuver was observed in the case of inferior vena cava (IVC) maximal repletion (25). Conversely, under hypovolemic conditions, increased mean thoracic pressure could induce the compressive occlusion of the IVC at its distal portion, at the junction with the right atrium, and lead to an acute cardiovascular collapse (26). Sympatho-vagal tone drives tolerance for acute intra thoracic pressure variation as it provides immediate inotropic, lusitropic and chronotropic adaptation (27).

Regarding the LV function, LV + dP/dtmax and shortened LV relaxation duration were observed in the conventional protective group, which can be explained by both higher ΔP_L_ in relation to probable overdistention and more marked sympathetic stress in this group. In line with the above-mentioned literature, our results suggest that TCAV might be safe assuming the IVC repletion. Further studies are needed to assess hemodynamic safety underlying increased mean thoracic pressures during prolonged periods of ventilation.

These results are in line with data from an existing animal sepsis model, with a less robust cardiac assessment, in which TCAV was safe compared with low tidal volume ventilation, in terms of CO and MAP. Further studies are needed to evaluate TCAV in other injury models (28).

### Respiratory Assessment of TCAV

The higher mean airway pressure and the lower respiratory rate observed in the TCAV group compared to the conventional protective group are explained by a longer I/E ratio, which is one of the fundamental characteristics of TCAV. Total PEEP was also higher, in relation with the decrease in ΔP_aw_ and improvement in El_L_. Tidal volume delivered in the TCAV group was closely monitored and averaged 7 mL/kg as T_low_ was adjusted to terminate at 75% of PEFR, in order to prevent alveolar collapse (7). P_L_Er provides indirect information about overdistension in the non-dependent lung areas and was lower at T_60_ in the TCAV group. TCAV significantly improved ΔP_aw_ and EIT regional compliance at T_60._This can be explained by a gain in aerated lung tissue volume. There were no differences between the two groups regarding both PaCO_2_ and pH values. Our results are in line with the literature, suggesting benefits of TCAV in terms of lung protective ventilation (12, 29).

### Study Limitations

One of the limitations of our study lies in the small sample size of each study group. The study might have been underpowered in its attempt to assess a clinically relevant effect of TCAV on hemodynamics. It is worth mentioning that dorsal decubitus is poorly tolerated in pigs and involves important modifications in both “West physiology” and hemodynamics that could mitigate external validation of the present results. Improvement in pulmonary elastance in the TCAV group can be in relation with higher levels of total PEEP and mean airway pressure. Furthermore, it could be suggestive of alveolar recruitment, but we did not perform any CT scan in order to verify this hypothesis, especially with the use of an recruitable ARDS (29, 30). In our work, the right ventricular function was assessed only with measures obtained with a pulmonary arterial catheter, as placement of the conductance catheter in the right ventricle and transthoracic echocardiography in pigs was not feasible in our study setting. The addition of paralysis may not fully encompass the hemodynamics associated with either ventilator mode as it does not incorporate the hemodynamic and respiratory effects of spontaneous breathing (28). To finish, this study was designed with only a 1-h ventilation period to observe the safety of initiation of TCAV on heart-lung interactions, limiting the evaluation of a longer period of TCAV on lung mechanics (31).

## Conclusion

In conclusion, no hemodynamic adverse events were observed with TCAV compared to standard protective ventilation in this swine ARDS model, as TCAV appeared to be beneficial for the respiratory system.

## Data Availability Statement

The original contributions presented in the study are included in the article/Supplementary Material, further inquiries can be directed to the corresponding author.

## Ethics Statement

The animal study protocol was approved by Nancy University Ethics Committee for Animal Experimentation (APAFIS Number 2020082407561244).

## Author Contributions

ML, BP, BL, MK, HP, and N'GT contributed to conception and design of the study. N'GT organized the study. BP and ML wrote the first draft of the manuscript. LB, J-LH, J-CR and MK wrote sections of the manuscript. All authors contributed to manuscript revision, read, and approved the submitted version.

## Funding

This research was funded by INSERM 1116, Région Grand Est, and FEDER.

## Conflict of Interest

The authors declare that the research was conducted in the absence of any commercial or financial relationships that could be construed as a potential conflict of interest.

## Publisher's Note

All claims expressed in this article are solely those of the authors and do not necessarily represent those of their affiliated organizations, or those of the publisher, the editors and the reviewers. Any product that may be evaluated in this article, or claim that may be made by its manufacturer, is not guaranteed or endorsed by the publisher.

## Abbreviations

APRV: Airway pressure release ventilation
ARDS: Acute respiratory distress syndrome
ATC: Automatic tube compensation
CO: Cardiac output
CPAP: Continuous positive airway pressure
C_RS_: Compliance of respiratory system
E_L_: Elastance of the lung
ER: Elastance ratio
E_RS_: Elastance of respiratory system
EtCO_2_: End-tidal carbon dioxide
EIT: Electrical Impedance Tomography
FIO_2_: Fraction of oxygen inspired
HiFi: High-fidelity pressure catheter
I:E: inspiratory to expiratory time ratio
IVC: Inferior vena cava
LVV: Left ventricular volume
PAWP: Pulmonary artery wedge pressure
P_aw_: Airway pressure
P_es_: Esophageal pressure
PBW: Predicted body weight
PEEP: Positive end-expiratory pressure
PEFR: Peak expiration flow rate
P_high_: High pressure
P_L_: Transpulmonary pressure
P_L_ER: Transpulmonary pressure according to ratio of elastance method
P_low_: Low pressure
PLV: Left ventricular pressure
P_es_: Pleural pressure or esophageal pressure
PVR: Pulmonary vascular resistance
RAP: Right atrial pressure
RR: Respiratory rate
S/D/M ABP, Systolic, diastolic: mean aortic blood pressure
S/D/M CBF, Systolic, diastolic: mean carotid blood flow
S/D/M PBP, Systolic, diastolic: mean pulmonary blood pressure
SVO_**2**_: Mixed venous oxygen saturation
TCAV: Time-controlled adaptive ventilation
T_high_: Time high
T_low_: Time low
VCV: Volume-controlled ventilation
VD: Dead volume
VILI: Ventilator-induced lung injury
LVV: Left ventricular volume
VT: Tidal volume
VV-ECMO: Veno-venous extracorporeal membrane oxygenation
ΔPaw: Driving pressure
ΔP_L_: Inspiratory transpulmonary pressure—expiratory transpulmonary pressure
ROI: Region of interest
RC_ROI_: Regional compliance.
