# Supplementary material for: Time-Controlled Adaptive Ventilation Does Not Induce Hemodynamic Impairment in a Swine ARDS Model
Source: Front Med (Lausanne). 2022 May 17;9:883950. doi: 10.3389/fmed.2022.883950 (PMC9152423; doi:10.3389/fmed.2022.883950)
Supplement: Supplementary file 1 [file Data_Sheet_1.docx]

**Supplementary Table 1**: Respiratory mechanics and hemodynamics at baseline and after ARDS induction in all animals

|  | Baseline measurements (n = 10) | ARDS status T0 (n = 10) |
| --- | --- | --- |
| Weight (kg) | 67 (63 – 73) | 67 (63 – 73) |
| Temperature (°C) | 38.0 (37.0 – 38.1) | 37.8 (37.0 – 38.5) |
| Hemoglobin (g.dL^-1^) | 9.7 (9.4 – 9.8) | 9.7 (9.7 – 11.5) |
| **Arterial blood gas** |  |  |
| pH | 7.41 (7.37 – 7.43) | 7.4 (7.32 – 7.46) |
| PaO**_2_** (mmHg) | 425 (328 – 487) | 98 (65 – 147) |
| PaCO**_2_** (mmHg) | 43 (41 – 46) | 37 (32 – 47) |
| SaO**_2_** (%) | 100 (100 – 100) | 96 (93 – 97) |
| Lactate (mmol.L^-1^) | 1.2 (1.0 –1.7) | 1.4 (1.0 –1.6) |
| Pa_O2_/Fi_O2_ ratio (mmHg) | 425 (328 – 487) | 99 (88 – 115) |
| **Settings of volume assist control** |  |  |
| VT (mL.kg^-1^) | 6.7 (6.3 –7.0) | 5.9 (5.7– 6.0) |
| RR (/min) | 23 (21 – 24) | 26 (25 – 27) |
| **Ventilator monitoring** |  |  |
| PEEPtot (cmH₂O) | 5 (5 – 6) | 6 (5 – 6) |
| Plateau pressure (cmH₂O) | 16.5 (15.3 –19.0) | 25.0 (23.0 – 26.7) |
| Mean airway pressure (cmH₂O) | 11.5 (10.3 – 14.0) | 14.5 (13.3 – 15.8) |
| ∆P_AW_ (cmH**_2_**O) | 11.1 (11.0 – 11.8) | 20.0 (18.0 – 20.5) |
| P_L_ end-expiratory (cmH**_2_**O) | 0.5 (0.1 – 1) | -0.3 (-3.0 – -0.5) |
| P_L_ end-inspiratory (cmH**_2_**O) | 8.4 (7.0 – 9.2) | 15.4 (14.3 – 16.3) |
| ∆P_L_ (cmH**_2_**O) | 8 (7 – 9) | 14 (13 –15) |
| Plateau esophageal pressure (cmH_2_O) | 8 (7 – 9) | 9 (7 –10) |
| End-expiratory esophageal pressure (cmH_2_O) | 5 (4 – 6) | 5 (4 – 7) |
| ∆P_ES_ | 3 (3 – 4) | 3 (2 – 3) |
| El_RS_ (cmH2O.L^-1^) | 25 (24 – 25) | 41 (38 – 42) |
| El_CW_ (cmH2O.L^-1^) | 5 (4 – 8) | 10 (4 –12) |
| El_L_ (cmH2O.L^-1^) | 20 (19 – 21) | 32 (29 – 33) |
| ER : El_L_/El_RS_ | 0.60 (0.58 – 0.61) | 0.81 (0.78 – 0.83) |
| Dead space (% of tidal volume) | 13.7 (11.4 – 16.0) | 28.2 (25.0 – 34.0) |
| **Hemodynamic parameters** |  |  |
| Heart rate (min^-1^) | 104 (94 – 122) | 148 (89 – 170) |
| Mean aortic blood pressure (mmHg) | 99 (79 –107) | 97 (83 –103) |
| Mean carotid blood flow (mL.min^-1^) | 203 (188 – 213) | 204 (194 – 210) |
| Systolic pulmonary blood pressure (mmHg) | 34 (29 – 36) | 50 (48 – 54) |
| Mean pulmonary blood pressure (mmHg) | 24 (20 – 25) | 38 (34 – 41) |
| Diastolic pulmonary blood pressure (mmHg) | 17 (15 –18) | 22 (20 – 28) |
| Right atrial pressure (mmHg) | 8 (6.25 – 8.75) | 9.5 (8.6 – 10.7) |
| PAWP (mmHg) | 9 (8 –11) | 11 (10 –12) |
| Pulmonary vascular resistance (U Woods) | 1.6 (1.3 –1.9) | 2.9 (2.3 – 4.1) |
| **Left ventricular function** |  |  |
| Cardiac output (L.min^-1^) | 5.20 (5,05 – 7,58) | 6.56 (5.23 – 9.46) |
| LV Tau_LV_ **_1/e_ (**ms) | 15.6 (14 – 17.11) | 15.6 (13.72 – 0.52) |
| LV +/– dP ratio | 1.01 (0.65 – 1.53) | 1.4 (0.8 – 2.7) |

Abbreviations: ∆P_AW_: driving pressure, difference in airway pressure at end-inspiration (plateau pressure) and end-expiration (total PEEP); P_L_ : Transplumonary pressure ; ∆P_L_ : difference in transpulmonary inspiratory pressure at end-inspiration and end-expiration ; ∆P_ES_ : difference in esophageal pressure at end-inspiration and end-expiration; El_CW_ : chest wall elastance; ER : Elastance ratio ; El_L_: lung elastance; El_RS_: respiratory system elastance; RR : Respiratory rate ; PEEPtot : Positive End Expiratory Pressure total; PAWP : Pulmonary artery wedge pressure ;Left Ventricular (LV) dP ratio represents LV catecholaminergic impregnation and was calculated as the ratio of LV +dP/dtmax and LV -dP/dtmax; Tau_LV_: Isovolumic relaxation time constant of the LV T0 : After ARDS induction ; T15 : 15 minutes after start of study ; T60 : 60 minutes after start of study ; Data are presented as median (25th – 75th percentile

**Supplementary Table 2 :** Additional hemodynamic characteristics in each study group over time.

|  | **TCAV (n=5)** | **Conventional protective ventilation (n=5)** | **Effect of group** | **Effect of time** | | **Group × time** | |  |
| --- | --- | --- | --- | --- | --- | --- | --- | --- |
| Systolic aortic blood pressure (mmHg) |  |  | p=0.6 | p=0.5 | | p=0.3 | |  |
| T0 | 121 (114 –124) | 123 (104 – 125) |  |  | |  | |  |
| T15 | 122 (114 – 117) | 110 (105 – 114) |  |  | |  | |  |
| T60 | 126 (124 –128) | 116 (112 – 117) |  |  | |  | |  |
| Diastolic aortic blood pressure (mmHg) |  |  | p=0.4 | p=0.8 | | p=0.4 | |  |
| T0 | 84 (77 – 88) | 70 (65 – 82) |  |  | |  | |  |
| T15 | 87 (82 – 91) | 72 (56 – 74) |  |  | |  | |  |
| T60 | 88 (74 – 97) | 70 (60 – 72) |  |  | |  | |  |
| Systolic carotid blood flow (mL.min^-1^) |  |  | p=0.3 | **p<0.05** | | p=0.4 | |  |
| T0 | 300 (295 – 320) | 347 (312 – 410) |  |  | |  | |  |
| T15 | 240 (232 – 245) | 292 (267 – 341) |  | # | |  | |  |
| T60 | 267 (220 – 300) | 259 (255 – 268) |  | # | |  | |  |
| Diastolic carotid blood flow (mL.min^-1^) |  |  | p=0.5 | **p<0.05** | | p=0.4 | |  |
| T0 | 136 (125 – 140) | 159 (155 – 160) |  |  | |  | |  |
| T15 | 105 (95 – 110) | 110 (100 – 125) |  | # | |  | |  |
| T60 | 70 (65 – 100) | 93 (85 – 105) |  | # | |  | |  |
| Mean carotid blood flow(mL.min^-1^) |  |  | p=0.9 | | **p<0.05** | | p=0.3 | |
| T0 | 197 (193 – 210) | 205 (203 – 220) |  | |  | |  | |
| T15 | 140 (128 – 160) | 169 (145 – 192) |  | |  | |  | |
| T60 | 170 (140 – 185) | 134 (120 – 150) |  | | # | |  | |
| Systolic pulmonary blood pressure (mmHg) |  |  | p=0.6 | p=0.1 | | p=0.8 | |  |
| T0 | 49 (44 – 55) | 52 (46 – 54) |  |  | |  | |  |
| T15 | 45 (43 – 49) | 47 (39 – 53) |  |  | |  | |  |
| T60 | 51 (45 – 54) | 50 (48 – 56) |  |  | |  | |  |
| Diastolic pulmonary blood pressure (mmHg) |  |  | p=0.3 | p<0.05 | | p=0.3 | |  |
| T0 | 28 (21 – 29) | 21 (18 – 22) |  |  | |  | |  |
| T15 | 27 (26 – 35) | 36 (25 – 39) |  |  | |  | |  |
| T60 | 33 (20 – 35) | 32 (27 – 42) |  | # | |  | |  |
| LV Ped(mmHg) |  |  | p=0.3 | p=0.2 | | p=0.4 | |  |
| T0 | 20 (15 – 23) | 16 (14 – 19) |  |  | |  | |  |
| T15 | 14 (10 – 18) | 13 (8 – 14) |  |  | |  | |  |
| T60 | 14 (11 – 15) | 10 (8 – 12) |  |  | |  | |  |
| Pmax (mmHg) |  |  | p=0.6 | p=0.5 | | p=0.3 | |  |
| T0 | 130 (120 –141) | 123 (104 – 137) |  |  | |  | |  |
| T15 | 142 (127 – 199) | 133 (103 – 139) |  |  | |  | |  |
| T60 | 113 (101 – 147) | 121 (111 – 128) |  |  | |  | |  |

The analysis used all data collected in both groups at the 3 study time points, using a mixed effects linear regression with study group and study time point as independent variables, and animal identification number as the random effect. Interaction of time with study group was systematically checked for. If no interaction was identified, the p value of the effect of Group and Time are given, respectively. In case of a significant interaction, a pairwise post-hoc multiple comparison was performed to compare groups at each time points on the one side, and compare T15 and T30 to T0 in each group, on the other.

#: p<0.05 compared to T0 at the time point (no interaction with study group)

Abbreviations: TCAV : Time controlled adaptative ventilation ; LV Ped : end-diastolic pressure ; Pmax : Maximum pressure during cardiac cycle. T0 : After ARDS induction ; T15 : 15 minutes after start of study ; T60 : 60 minutes after start of study ; Data are presented as median (25th – 75th percentile

**Supplementary Table 3** : Additional respiratory characteristics in each group over time.

|  | **TCAV (n=5)** | **Conventional protective ventilation (n=5)** | **Effect of group** | **Effect of time** | **Group × time** |
| --- | --- | --- | --- | --- | --- |
| **General characteristics** |  |  | |  | |
| Weight (kg) | 67 (66 – 74) | 67 (63 – 73) | - | - | - |
| ED (mm) | 7 (7 – 7) | 7 (7 – 7.5) | - | - | - |
| **I/E** |  |  | - | - | **p<0.05** |
| T0 | 1/2 (1/2 – 1/2.3) | 1/2 (1/1.8 – 1/2.4) |  |  |  |
| T15 | 1/7 (1/6 – 1/7)^a^ | 1/2.2 (1/1.9 – 1/2.3)^c^ |  |  |  |
| T60 | 1/7 (1/6 – 1/7)^a^ | 1/2.2 (1/1.9 – 1/2.3)^c^ |  |  |  |
| **Minute ventilation (L.min^-1^)** |  |  | - | - | **p<0.05** |
| T0 | 10.8 (10.0 –11.5) | 10.2 (9.7– 11.1) |  |  |  |
| T15 | 9.3 (8.3 – 10.3)^a^ | 11.5 (10.6 – 11.5)^bc^ |  |  |  |
| T60 | 9.2 (7.9 – 11)^a^ | 11.1 (10.5 – 11.8)^bc^ |  |  |  |
| **Plateau pressure (cmH₂O)** |  |  | - | - | **p<0.05** |
| T0 | 25 (23 – 26) | 25 (24 – 27) |  |  |  |
| T15 | 28 (27 – 29)^a^ | 26 (25 – 27)^bc^ |  |  |  |
| T60 | 28 (27 – 29)^a^ | 26 (25 – 27)^bc^ |  |  |  |
| **Mean airway pressure (cmH₂O)** |  |  |  |  |  |
| T0 | 14 (13 –16) | 15 (14 –16) | - | - | **p<0.05** |
| T15 | 25 (24 – 25)^a^ | 21 (21 – 22)^bc^ |  |  |  |
| T60 | 25 (25 – 26)^a^ | 22 (22 – 23)^bc^ |  |  |  |
| **Plateau esophageal pressure (cmH_2_O)** |  |  | p=0.30 | **p<0.05** | p=0.20 |
| T0 | 9 (6 – 11) | 10 (9 – 10) |  |  |  |
| T15 | 12 (11 – 12) | 11 (10 – 12) |  | # |  |
| T60 | 13 (12 –16) | 12 (11 – 13) |  | # |  |
| **End-expiratory esophageal pressure (cmH_2_O)** |  |  | p=0.20 | **p<0.05** | p=0.30 |
| T0 | 9 (6 - 11) | 10 (9 – 10) |  |  |  |
| T15 | 9 (8 - 10) | 9 (9 – 10) |  | # |  |
| T60 | 10 (10 –12) | 10 (10 – 12) |  | # |  |
| **P_L_ end-expiratory (cmH_2_O)** |  |  | - | - | **p<0.05** |
| T0 | -3 (-3 – 0) | 0 (-1 – 0) |  |  |  |
| T15 | 3 (3 – 4)^a^ | 1 (0 – 1)^c^ |  |  |  |
| T60 | 2 (2 – 5)^a^ | 0 (-1 – 1)^c^ |  |  |  |
| **P_L_ plateau pressure (cmH_2_O)** |  |  | **p<0.05** | p=0.82 | p=0.32 |
| T0 | 15 (12 – 16) | 15 (15 – 17) |  |  |  |
| T15 | 14 (13 – 15) | 19 (15 – 20) |  |  |  |
| T60 | 13 (12 – 14) | 17 (16 – 18) |  |  |  |
| **P_L_ plateau pressure ER (cmH_2_O)** |  |  | - | - | **p<0.05** |
| T0 | 24 (19 – 25) | 21 (18 – 24) |  |  |  |
| T15 | 19 (18 – 20)^a^ | 25 (24 – 30)^c^ |  |  |  |
| T60 | 18 (15 – 19)^a^ | 26 (25 – 27)^c^ |  |  |  |
| **El_rs_ (cmH₂O.L^-1^)** |  |  | - | - | **p<0.05** |
| T0 | 45 (44 – 46) | 49 (47 – 51) |  |  |  |
| T15 | 30 (20 – 32)^a^ | 47 (42 – 50)^c^ |  |  |  |
| T60 | 28 (18 – 31)^a^ | 45 (40 – 49)^c^ |  |  |  |
| **El_CW_ (cmH₂O.L^-1^)** |  |  | - | - | **p<0.05** |
| T0 | 4 (2 – 10) | 9 (8 – 11) |  |  |  |
| T15 | 6 (5 – 7) | 6 (2 – 9) |  |  |  |
| T60 | 6 (2 – 8) | 4 (3 – 5)^b^ |  |  |  |
| **El_L_/El_RS_** |  |  | p=0.51 | **p<0.05** | p=0.42 |
| T0 | 0.60 (0.55 – 0.62) | 0.61 (0.60 – 0.62) |  |  |  |
| T15 | 0.78 (0.64 – 0.79) | 0.83 (0.81 – 0.85) |  | # |  |
| T60 | 0.83 (0.80 – 0.84) | 0.86 (0.83 – 0.89) |  | # |  |
| **Dead space (% of tidal volume)** |  |  | p=0.71 | p=0.62 | p=0.32 |
| T0 | 26 (23 - 26) | 30 (29 - 35) |  |  |  |
| T15 | 17 (12 – 21) | 22 (15 – 24) |  |  |  |
| T60 | 15 (14 – 18) | 24 (11 – 31) |  |  |  |
| **SaO_2_** (%) |  |  | p=0.41 | **p<0.05** | p=0.31 |
| T0 | 95 (94 – 95) | 93 (93 – 94) |  |  |  |
| T15 | 98 (93 – 99) | 92 (90 – 94) |  |  |  |
| T60 | 96 (94 – 98) | 94 (85 – 97) |  | # |  |
| **pH** |  |  | - | - | **p<0.05** |
| T0 | 7.31 (7.30 – 7.34) | 7.37 (7.34 – 7.38)^c^ |  |  |  |
| T15 | 7.39 (7.35 – 7.40) | 7.38 (7.36 – 7.39) |  |  |  |
| T60 | 7.36 (7.34 – 7.40) | 7.31 (7.30 – 7.32) |  |  |  |
| **HCO3- (mmol.L^-1^)** |  |  | p=0.11 | p=0.22 | p=0.07 |
| T0 | 20 (18 – 21) | 22 (19 – 23) |  |  |  |
| T15 | 21 (20 – 22) | 21 (21 – 23) |  |  |  |
| T60 | 20 (19 – 22) | 22 (20 – 24) |  |  |  |
| **Hb (g/dL)** |  |  | p=0.41 | p=0.20 | p=0.31 |
| T0 | 9.4 (8.5 – 9.8) | 9.7 (9.7 – 9.8) |  |  |  |
| T15 | 9.9 (9.7 – 11.0) | 9.8 (9.0 – 11.0) |  |  |  |
| T60 | 9.8 (9.7 – 10.0) | 9.5 (9.2 – 9.1) |  |  |  |
| **Lactate (mol.L^-1^)** |  |  | p=0.11 | p=0.22 | p=0.06 |
| T0 | 1.1 (1.0 – 1.2) | 1.5 (1.1 – 1.8) |  |  |  |
| T15 | 1.1 (0.9 – 2.0) | 1.4 (1.3 – 1.5) |  |  |  |
| T60 | 1.1 (1.0 – 2.1) | 1.5 (1.5 – 1.7) |  |  |  |

The analysis used all data collected in both groups at the 3 study time points, using a mixed effects linear regression with study group and study time point as independent variables, and animal identification number as the random effect. Interaction of time with study group was systematically checked for. If no interaction was identified, the p value of the effect of Group and Time are given, respectively. In case of a significant interaction, a pairwise post-hoc multiple comparison was performed to compare groups at each time points on the one side, and compare T15 and T30 to T0 in each group, on the other.

#: p<0.05 compared to T0 at the time point (no interaction with study group)

a: p<0.05 compared to T0 in the TCAV group in multiple comparison

b: p<0.05 compared to T0 in the conventional protective ventilation group in multiple comparison

c: p<0.05 compared to the TCAV group at this time point in multiple comparison

Abbreviations: TCAV : Time controlled adaptative ventilation ; C_RS_: Respiratory static compliance. P_L_ : Transpulmonary pressure ; El_CW_ : chest wall elastance; El_RS_: respiratory system elastance. T0 : After ARDS induction ; T15 : 15 minutes after start of study ; T60 : 60 minutes after start of study ; Data are presented as median (25th – 75th percentile).

**Supplementary Table 4:** Electrical impedance tomography values in each group over time.

|  | **TCAV (n=5)** | **Conventional protective ventilation (n=5)** | **Effect of group** | **Effect of time** | **Group × time** |
| --- | --- | --- | --- | --- | --- |
| **ROI 1 ventral (%)** |  |  | - | - | **p<0.05** |
| T0 | 14 (11 - 21) | 16 (11 - 26) |  |  |  |
| T15 | 8 (6 - 12)^a^ | 10 (9 - 16) |  |  |  |
| T60 | 7 (6 - 9)^a^ | 9 (8 - 12)^b^ |  |  |  |
| **ROI 2 mid–ventral (%)** |  |  | p=0.6 | p=0.09 | p=0.8 |
| T0 | 63 (53 - 66) | 58 (53 - 63) |  |  |  |
| T15 | 58 (50 - 60) | 58 (51 - 60) |  |  |  |
| T60 | 54 (50 - 56) | 58 (46 - 59) |  |  |  |
| **ROI 3 mid-dorsal (%)** |  |  | - | - | **p<0.05** |
| T0 | 18 (14 - 21) | 20 (18 - 22) |  |  |  |
| T15 | 27 (25 - 32)^a^ | 27 (26 - 28)^b^ |  |  |  |
| T60 | 33 (31 - 35)^a^ | 27 (26 - 29)^bc^ |  |  |  |
| **ROI 4 dorsal (%)** |  |  | p=0.2 | p=0.1 | p=0.09 |
| T0 | 3 (2 - 4) | 4 (4 - 5) |  |  |  |
| T15 | 4 (5 - 6) | 4 (3 - 4) |  |  |  |
| T60 | 4 (3 - 5) | 4 (3 - 5) |  |  |  |
| **RC_ROI1_ ventral (ml/cmH_2_O)** |  |  | p=0.2 | p=0.4 | p=0.3 |
| T0 | 2.8 (2.2 - 4.8) | 2.7 (2.3 - 5.2) |  |  |  |
| T15 | 2.6 (2.0 - 4.8) | 2.3 (2.0 - 3.4) |  |  |  |
| T60 | 2.4 (3.4 - 5.1) | 2.3 (2.1 - 3.0) |  |  |  |
| **RC_ROI2_ mid-ventral (ml/cmH_2_O)** |  |  | - | - | **p<0.05** |
| T0 | 12.9 (11.0 - 13.2) | 11.6 (10.9 - 12.1) |  |  |  |
| T15 | 16.0 (14.0 - 21.8)^a^ | 12.4 (11.1 - 12.5) |  |  |  |
| T60 | 19.0 (15.7 - 25.1)^a^ | 10.0 (9.8 - 13.2)^c^ |  |  |  |
| **RC_ROI3_ mid-dorsal (ml/cmH_2_O)** |  |  | - | - | **p<0.05** |
| T0 | 2.8 (3.9 - 4.5) | 4.0 (3.9 - 4.6) |  |  |  |
| T15 | 8.6 (8.3 - 10.2)^a^ | 5.2 (5.0 - 6.3)^c^ |  |  |  |
| T60 | 10.2 (9.4 - 13.5)^a^ | 5.5 (5.1 - 6.2)^c^ |  |  |  |
| **RC_ROI4_ dorsal (ml/cmH_2_O)** |  |  | p=0.3 | p=0.5 | p=0.4 |
| T0 | 0.9 (0.8 - 1.1) | 1.0 (0.8 - 1.2) |  |  |  |
| T15 | 1.0 (0.7 - 1.2) | 0.8 (0.7 - 0.8) |  |  |  |
| T60 | 0.7 (1.2 - 1.9) | 0.8 (0.8 - 0.8) |  |  |  |

Data are expressed as median (quartile 1 – quartile 3). The analysis used all data collected in both groups at the 3 study time points, using a mixed effects linear regression with study group and study time point as independent variables, and animal identification number as the random effect. Interaction of time with study group was systematically checked for. If no interaction was identified, the p value of the effect of Group and Time are given, respectively. In case of a significant interaction, a pairwise post-hoc multiple comparison was performed to compare groups at each time points on the one side, and compare T15 and T30 to T0 in each group, on the other.

a: p<0.05 compared to T0 in the TCAV group in multiple comparison

b: p<0.05 compared to T0 in the conventional protective ventilation group in multiple comparison

c: p<0.05 compared to the TCAV group at this time point in multiple comparison

Abbreviations: TCAV : Time controlled adaptative ventilation ; ROI : region of interest ; RC_ROI_ : Regional compliance of the region of interest ; T0 : After ARDS induction ; T15 : 15 minutes after start of study ; T60 : 60 minutes after start of study ; Data are presented as median (25th – 75th percentile).

Supplementary Figure 1: Timeline of the study. Abbreviations: TCAV : Time controlled adaptative ventilation ; TB : Basal time after respiratory and hemodynamics monitoring T0 : After ARDS induction ; T15 : 15 minutes after randomization ; T60 : 60 minutes after randomization
